# Form Matters: Design Creativity in Positive Psychological Interventions

**DOI:** 10.1186/s13612-016-0043-5

**Published:** 2016-07-04

**Authors:** Pieter M. A. Desmet, Maria C. Sääksjärvi

**Affiliations:** Department of Industrial Design, Delft University of Technology, Landbergstraat 15, 2628 CE Delft, The Netherlands; Department of Product Innovation Management, Delft University of Technology, Landbergstraat 15, 2628 CE Delft, The Netherlands

**Keywords:** Design creativity, Behavioural intervention technologies, Positive psychology interventions

## Abstract

**Background:**

The current article explores the effect of design on the efficacy of behavioural intervention technologies (BITs). With a user-centred design process, colourful key ring coins were created as a means of introducing self-administered behavioural interventions.

**Methods:**

A 6-week study tested whether the tangible objects contributed to the effectiveness of these interventions. Three groups were compared (N = 100): one group received happiness-enhancing activities printed on key ring coins, one group received the same activity tasks printed on paper, and one group served as a control. The outcome measure was the satisfaction with life scale (SWLS).

**Results:**

The group that received happiness-enhancing activities on key ring coins scored highest on SWLS. Participants mentioned that it was exciting to be reminded to do the task whenever they were using their keys. Others mentioned that the coins helped them to put their hearts into the project, trying their best to finish the tasks.

**Conclusions:**

The findings support the proposition that design should be recognized as an important factor when developing effective means for disseminating positive psychology to a broad audience. This highlights the need for multidisciplinary approaches to the development of BITs, embracing active collaborations between psychologists, computer scientists, and (interaction) designers.

## Background

Research in the field of positive psychology has provided substantial evidence that individuals can increase and sustain their happiness (Lyubomirsky 2008; Diener 1999) and that the best way to do so is by aligning one’s behaviours with those of people who flourish (for an overview, see Schueller and Parks 2014). Inspired by these findings, a wide variety of interventions have been introduced that aim to help people to actively pursue greater levels of happiness (Parks and Biswas-Diener 2013). These so-called positive psychology interventions (PPIs) have been described as ‘treatment methods or intentional activities aimed at cultivating positive feelings, positive behaviours, or positive cognitions’ (Sin and Lyubomirsky 2009, p. 468). PPIs are very diverse, including intentional activities such as writing gratitude letters, learning to forgive, and taking care of one’s body. Based on a literature review, Schuller and Parks (2014) identified five broad categories of evidence-based interventions: (1) savouring experiences and sensations, (2) cultivating gratitude, (3) engaging in kind acts, (4) promoting positive relations, and (5) pursuing hope and meaning. Although the study of PPIs is relatively new, there is growing evidence that they are effective in boosting long-term wellbeing (see two recently published meta-analyses: Bolier et al. 2013; Sin and Lyubomirsky 2009).

While traditional PPIs require a one-to-one interaction between a client and a coach or therapist, innovative modes of distribution can promote the flourishing of a broad population. One interesting direction is through self-help—providing resources directly to those who are interested without professional assistance (Parks 2015; Schueller and Parks 2014). Traditionally, such self-administered PPIs have reached an audience through self-help books. More recently, several technology-based interventions have been introduced. These behavioural intervention technologies (BITs) use digital media to introduce PPIs to a general public. A well-known example is the Live Happy iPhone app (USA, Signal Patterns) that was based on the work of Lyubomirsky (2008) and contains a variety of exercises, such as savouring the day, doing kind things, and making a gratitude journal. Another example is Psyfit (NL, Trimbos Institute), an online unguided self-help intervention that consists of six modules with four lessons each, which users can tailor to meet their personal needs.

Automated self-help BITs are scalable to reach hundreds of thousands of people worldwide (Muñoz 2010). They can be used repeatedly without losing their therapeutic power to help additional people and they can transcend space, time, culture, and language because they can be used simultaneously anywhere in the world, at a time of the person’s choosing. BITs can engage and empower participants to take charge of their own well-being; they can stimulate self-management skills by providing participants with tools to guide their behaviour, thoughts, and interactions (Bolier 2015). While this also applies to self-help books, BITs have several additional advantages. They can help in translating acquired insights into real life and provide support to users in devoting the time and effort required for sustainable behavioural change (McGonical 2011). BITs can be deeply integrated into people’s daily lives. As BITs are provided on devices that people access and carry throughout their day (such as computers, tablets, smartphones, and wearable computing), they can increase the user’s awareness in times, places, and situations to help cope with their daily issues (Schueller et al. 2013).

While BITs are increasingly recognized as promising means for disseminating positive psychology to a broad audience, Schueller et al. (2013) identified some key obstacles that should be overcome for BITs to realize their full potential. One of these obstacles is a general shortage of innovativeness in using the possibilities of technology. Many BITs are designed to mimic regular therapy-based PPIs. They are structured as sessions for weekly use and employ text to introduce interventions. BITs do not need to be constrained by such traditional structures, and (interaction) design is crucial for unlocking the possibilities for developing BITs that are effective and engaging. This implies that BITs will realize their full potential only with deliberate and thoughtful design. Design science offers several domains of knowledge that can help in developing successful BITs. These include traditional user-centred design approaches, co-design, experience design, gamification, usability, and aesthetics, and also recent developments in design research, such as design for subjective well-being (Desmet and Pohlmeyer 2013; Hassenzahl 2010) and positive computing (Sander 2011; Calvo and Peters 2014).

In this paper, we introduce a BIT that was developed with a user-centred design approach. The aim is to illustrate that BITs do not necessarily have to be restricted to screen-based interactions because design creativity can open up a broader notion of technology. The designed BIT uses tangible coins as a means for bringing activity interventions into the daily lives of users. We report a six-week study that tested the added value of tangible coins over a more traditional written means. This study provided evidence for the proposition that ‘form matters’: design qualities influence the effectiveness of a BIT. This finding supports the view that (a) design should be embraced as an important means for increasing the impact of future BITs, and that (b) exploring how BITs can optimally integrate digital and non-digital technology is a promising direction.

## Design-Driven Positive Intervention: TinyTask

TinyTask is a BIT that uses tangible means to stimulate people to engage in happiness-increasing activities. The design was based on the ‘sustainable happiness model’ of Lyubomirsky et al. (2005), which proposes that the most effective way to increase and maintain one’s chronic happiness over and above the genetic set point is by changing one’s behaviour via intentional activities. These include activities that are behavioural (e.g. being kind to others), cognitive (e.g. counting one’s blessings), or volitional (e.g. devoting effort to meaningful causes). Shelden and Lyubomirsky (2006) proposed that such activities could combat the effects of hedonic adaptation because they are episodic (instead of continuous) and can be varied in terms of timing, approach, and content. As a consequence, intentional activities can have lasting benefits for our well-being (Lyubomirsky 2008; Layous and Lyubomirsky 2014; Lyubomirsky 2001). TinyTask was based on the 12 intentional activities detailed in the self-help book *The How of Happiness* (Lyubomirsky 2008), which have been recently shown to be effective for increasing life-satisfaction (Parks and Szanto 2013).

The TinyTask designer aimed to design a BIT that was (1) enabling, motivating, and engaging, and (2) at the same time unobtrusive, discreet, and aesthetically pleasing. These intentions were informed by two challenges. The first was that engaging in intentional activities is not necessarily easy. A person needs to ‘get over the hurdle’ of remembering to do them and overcome obstacles in initiating them, and this kind of self-regulatory effort requires considerable self-discipline and willpower (Sheldon and Elliot 1998; Lyubomirsky et al. 2005). In other words, effectively initiating and pursuing happiness-increasing activities requires commitment and effort. As a consequence, people often need external support to be able to invest the time and energy necessary for sustainable behavioural change, and to effectively translate acquired knowledge into practice (Bolier 2015). The second challenge was that people will only want to use BITs if they provide acceptable levels of privacy and are unobtrusive, aesthetically pleasing, and trustworthy; people do not accept BITs that rely on the use of complicated, invasive, or demanding interfaces (Consolvo et al. 2009; Montague et al. 2009).

Lyubomirsky et al. (2005) offered two suggestions to help people get over the hurdle of engaging in intentional activities. The first is to start with those activities that are intrinsically more appealing than others, and the second is to create a habit out of regularly initiating beneficial activities. These suggestions partly overlap with three conditions for successfully changing behaviour (i.e. motivation, ability, and triggers) that were proposed by Fogg (2009): people can only change their behaviour when they are motivated to do so, when they are able to translate their motivation into concrete action, and if there are well-timed triggers to initiate new behaviour. The findings of Parks et al. (2012) were also taken into consideration in the design process. They found that people get bored performing the same happiness-enhancing activities, and that happiness is improved more when people engage in a greater variety of activities. All these authors demonstrate the crucial role of *intrinsic motivation* in the development of effective BITs. Intrinsic motivation refers to doing something because it is inherently interesting or enjoyable, as opposed to being motivated by separable rewards or external pressures (Ryan and Deci 2000). These are activities that people do for the enjoyment of the activity itself, due to their appeal of novelty, challenge, or aesthetic value. Moreover, intrinsic motivation is facilitated by environments that support feelings of autonomy and competence (see Deci and Ryan 1985). Given these insights, TinyTask was designed with the intention to foster intrinsic motivation by integrating five key qualities:

1. Distil a range of general and abstract strategies into smaller (tiny), comprehensible tasks.
2. Provide an immediate sense of pleasure and achievement while (and after) fulfilling the task.
3. Offer several tangible and concrete triggers to stimulate the intended activities.
4. Offer a simple structure that enables people to form a habit of engaging in the activities.
5. Present a broad diversity of activities to stimulate interest, to keep the experiences fresh and to offer a sense of choice.

**Fig. 1:**
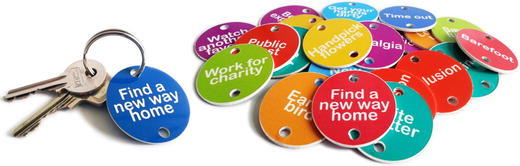
Key ring coins

The result is a set of incrementally distributed, colourful key ring coins (Fig. 1), which was developed in an iterative design process, involving end-users in several stages of the project (for a report on the development, see Ruitenberg 2011). Every coin represents a small task, or ‘tiny task’, the accomplishment of which employs one of the twelve happiness strategies via a concrete, low threshold activity.

**Fig. 2:**
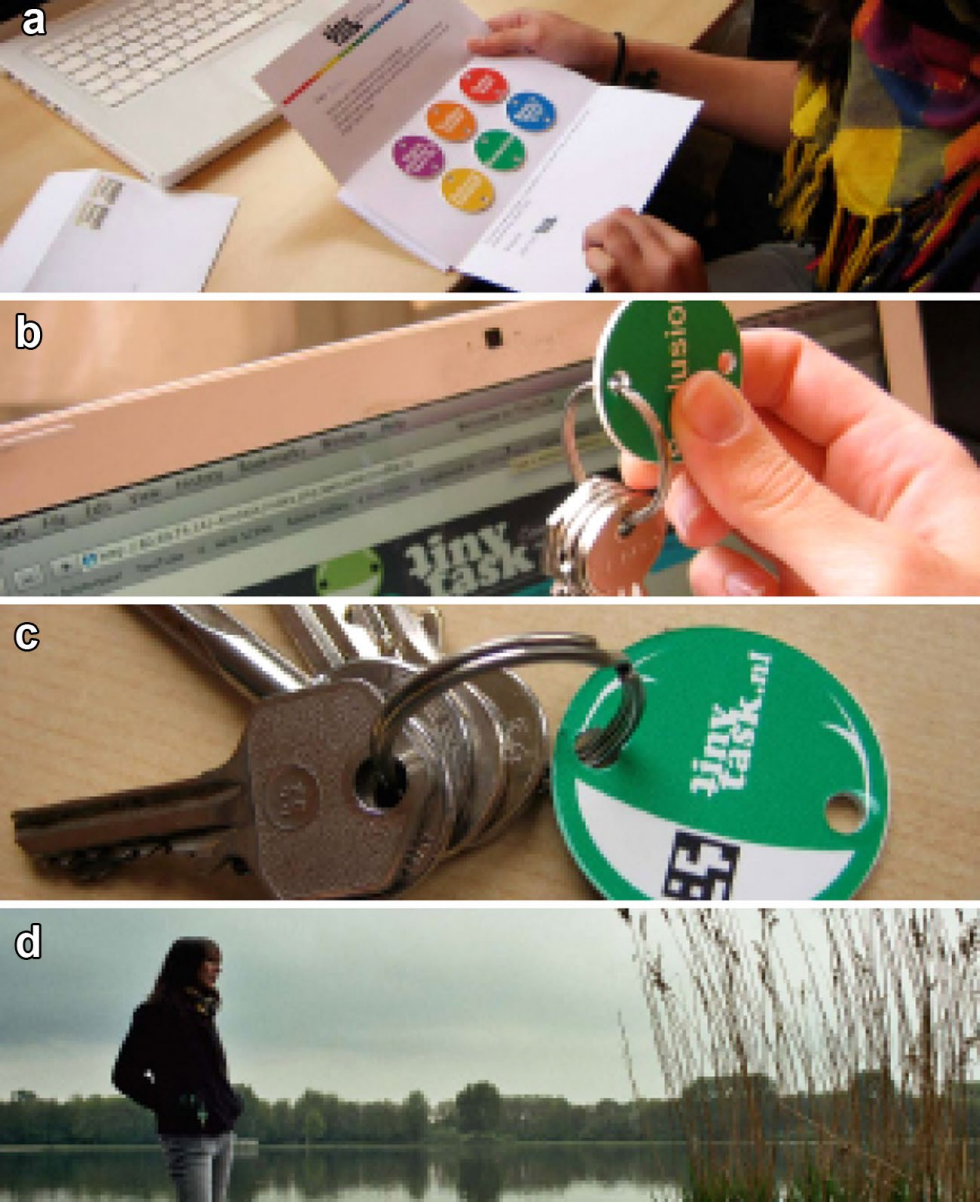
TinyTask usage scenario. **a** Receiving ring coins; **b** select one and read task description online; **c** commit by connecting to keychain; **d** do the task

Figure 2 visualizes a basic usage scenario. Once registered, the user receives an initial envelope with six key ring coins (Fig. 2a). Each coin face bears an inscription hinting at what the assignment is about, such as: ‘early bird’, ‘improvise your meal’, or, as in the example shown in Fig. 2, ‘seclusion’. On the back of the coin is a symbol. With this, the user can locate the full assignment on the TinyTask website (Fig. 2b; for the ‘seclusion’ example: ‘Find a place in nature that is secluded from traffic and buildings. Listen to the birds, wind, and surroundings.’). Users select one coin to commit to, and then attach the coin inscribed with the chosen task to their key ring (Fig. 2c). By attaching the coin, they commit to the task, and are reminded about that commitment every time they pick up their keys. Once the task has been performed (Fig. 2d), users can remove the coin (and save it or give it to a friend) and attach another one. When five out of six assignments are completed, the user will receive a new set of coins.

The assignments were formulated to be small, concrete, and original, ensuring a high level of capability. Users express their commitment to an assignment by attaching the related coin to their key rings, where it serves as a reminder of the commitment. The act of selecting a coin, attaching it to the key ring, and removing it once the task has been done is enjoyable and implicitly rewarding, increasing the motivation of users to engage in the behaviour. Moreover, the system of sending one small fresh set of coins at a time also increases motivation, because receiving a new envelope is like a small and exciting gift.

### TinyTask Activities

An important step in the development was to translate the general happiness strategies into small (tiny) activities that could be implemented through everyday events. As a first step, the 12 categories were used as the basis for a brainstorming session with eight participants (four male, four female). This session generated an initial list of 243 activities. At the end of the brainstorm session, this list was reduced to 70 activities, according to the criteria of viability (i.e. the activity does not require special skills or tools), appeal (i.e. the activity is expected to be liked by a wide variety of people), and variety (i.e. very little overlap between activities). The second step in the development process was a pre-test. Over a period of 3 weeks, a group of 15 volunteers (six women, nine men; aged 21–60) tested the 70 activities. After the testing period, participants were interviewed to obtain feedback about their experiences. These results were used to reduce the set of activities further to a final set of 50 happiness-enhancing activities. As a final step, these 50 activities were reviewed by two experts in the field (one healthcare expert and one expert in health promotion). On the basis of their comments, some minor additional modifications to the activities were made. The final list of happiness-enhancing activities can be found in Appendix 1.

## Main Study

The study was designed to empirically test the happiness effects obtained by using TinyTask. Our proposition was that it contributes to user happiness, not only thanks to the (intangible) activities that it stimulates, but also because of the material design qualities of the (tangible) key ring coins. In order to test this dual-natured proposition, we designed a six-week longitudinal experiment. Our two main hypotheses were that (a) engaging in happiness tasks has a positive effect on happiness, and that (b) when these tasks are communicated with physical key ring coins, the happiness effect is greater than when they are communicated with conventional means (written on paper). An additional aim of the study was to explore the timing of new task distribution. In the literature on PPIs, it is not yet known what kind of distribution frequency is optimal (i.e. distribute evenly or in batches). For optimization purposes, a distribution condition was included in the study. Three research questions were formulated:

1. Does performing the TinyTask assignments have an effect on the happiness of participants, compared to a control group that receives no assignments?
2. Do the physical key ring coins have an effect on the happiness of participants, compared to a group that receives the assignments on paper?
3. Does the timing of the tasks influence the happiness of participants? Is an intervention with a group that receives one task per day of the week more effective in generating happiness than an intervention with a group that receives five tasks at the start of each week?

### Participants

The participant cohort was composed of 100 volunteer students enrolled in a major university in the Netherlands. They were recruited using in-class announcements, flyers, and social media. During the recruitment campaign, the general topic (happiness) of the study was mentioned. Before making their final decision regarding whether to participate in the study or not, prospective participants were informed of the study’s longitudinal nature, and told that they would need to set aside at least 15 min every day to perform specific activities and answer questionnaires. Participants’ ages ranged from 18 to 32 (M = 23; SD = 3.53) and the sample included the same number of females as males (50 men, 50 women). After completing the study, each participant was given a 20-euro gift voucher and an informative booklet about strategies to improve happiness as a token of gratitude for their contribution. The results of three participants were not included in the analysis because they had failed to complete all questionnaires.

### Design

We made a 2 × 2, between-subject experimental design (see Table 1). The first independent variable was ‘design’, with two levels: happiness activities written on paper (Paper) versus happiness activities printed on a key ring coin (Coins). The second independent variable was ‘intensity’, with two levels: one task a day for 5 days (Spread) versus five tasks on 1 day (Condensed). These variables combined to form four experimental groups. In line with other studies on happiness activities (e.g. Lyubomirsky et al. 2011), we also had a control group whose members did not engage in happiness-enhancing activities.

**Table 1:** Four experimental groups

Intensity	Design	
	Coin	Paper
Spread (one task a day)	A1	B1
Condensed (five tasks on 1 day)	A2	B2

Participants were randomly assigned to one of the five groups. All participants (except those in the control group) received six happiness-enhancing activities (from here on called ‘tasks’) per week, for 5 weeks. The order and selection of tasks was randomized for each participant. Task selection was balanced so that each of the 50 tasks in Appendix 1 was given to the same number of participants, meaning that every participant received a randomized selection (30) of the total number of tasks (50). Participants in Groups B1 and B2 (Paper) received the tasks printed on paper. This is consistent with how happiness-enhancing activities are typically administered in the well-being literature (see Sin and Lyubomirsky 2009). Participants in Groups A1 and A2 (Coin) received the tasks printed on coins, which were made of brightly coloured hard plastic (see Fig. 2). Participants in Groups A1 and B1 (Spread) received the tasks spread out over the week: one task to be performed every day (except on Sunday). Participants in Groups A2 and B2 (Condensed) received five tasks on the first day of the week; all tasks to be performed in 1 day. Respondents received their tasks in batches of six, in neutral white envelopes.

### Procedure

The study was conducted over a time period of 6 weeks. In the first week, the participants filled out a pre-study questionnaire. In each of the remaining 5 weeks, all participants (except those in the control group) performed six tasks. They were instructed to spend as much time on performing the activities as they wished. In line with typical procedures followed by previous studies on happiness-enhancing activities, participants in the control group were asked to reflect on a daily event and write about it in a journal (for the procedure, see Sin and Lyubomirsky 2009). Data were collected on a daily and weekly basis, following the procedure of Lyubomirsky et al. (2011). Each day, participants answered a short questionnaire. They filled in a longer questionnaire on a weekly basis. In addition, they filled out one questionnaire before the start of the study (benchmark) and one at the end of the study. Participants in the control group responded to the same number of questionnaires as those who completed the happiness activities.

### Measures

Following common practice in positive psychology intervention studies, we measured the well-being effect with the satisfaction with life scale (SWLS), a short 5-item method that requires about 1 min to fill out (Diener et al. 1985; Pavot et al. 1991). The pre-study questionnaire included general questions about age, gender, and nationality, and the first SWLS measurement. In the daily questionnaire, participants reported on the activity they had completed on that particular day. As it is common practice in positive psychology intervention studies to control for enjoyment and effort, participants reported how much they enjoyed doing that activity and how much effort they had put into the activity (on 7-point scales). The Sunday questionnaire was similar, but also measured SWLS and asked the respondents to report what task they had enjoyed the most that week, and to what degree they would like to do that task again in the future (on a 7-point scale). Participants in the control group filled out similar daily and weekly questionnaires in which questions about activities were changed to questions about the daily event that they wrote about in their journal.

## Results

**Fig. 3:**
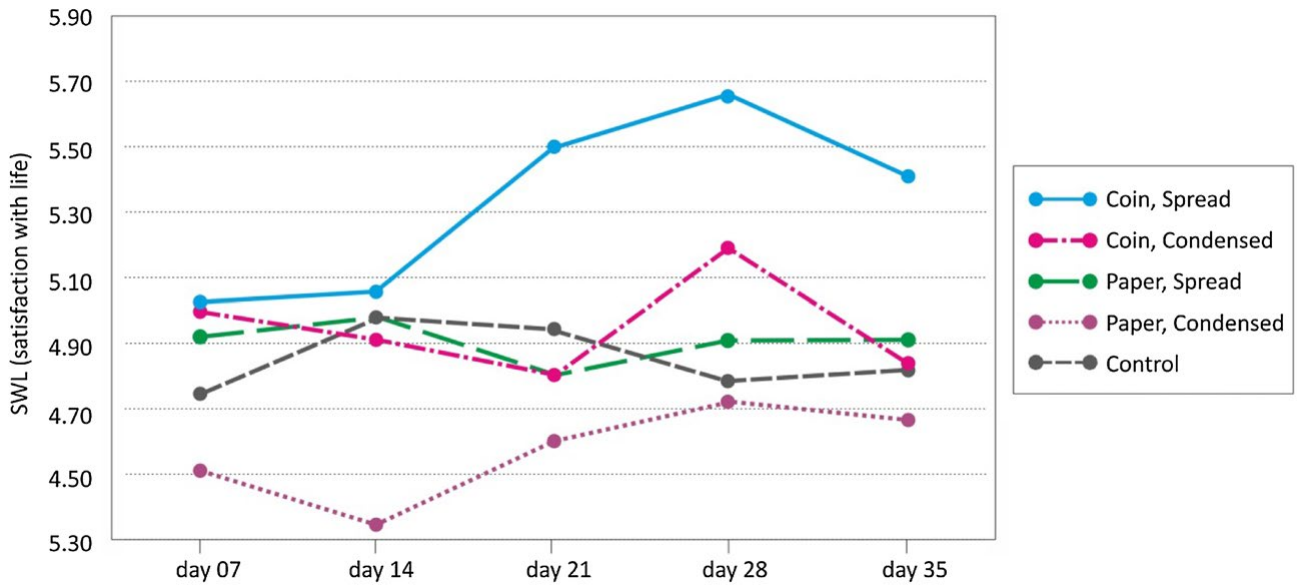
Satisfaction with life (SWL) means over time

To examine the research questions, we conducted a 3 (condition: control, coin, paper) × 2 (intensity: spread, condensed) mixed ANOVA with condition and intensity serving as between-subjects measures and SWL as a within-subjects measure. Our results revealed a significant three-way effect between condition, intensity, and SWL [F(4, 324) = 4.31, p = 0.002 (all other effects were non-significant: main effect of condition F(1, 81) = 0.49, p = 0.484, main effect of pacing F(1, 81) = 2.18, p = 0.144, main effect of SWL F(4, 324) = 2.23, p = 0.065, condition x pacing F(1, 81) = 0.01, p = 0.981, condition x SWL F(4, 324) = 0.63, p = 0.642, pacing x SWL F(4, 324) = 0.89, p = 0.469; see Table 2 for means and SDs)]. Including enjoyment and effort as covariates showed that participants’ enjoyment and effort varied over time (significant interaction between time and enjoyment and effort; F = 2.612, p = 0.036), but, consistent with expectations, it did not influence our experimental manipulation or its link to SWL. Our results show that participants who received coins in a spread out fashion reported the highest SWL level, and that their SWL, in general, increased over time. This effect can also be seen in Fig. 3 with the overall SWL level increasing until the last week when it starts to taper off, probably due to fatigue effects that are typically encountered in longitudinal studies. Note that it is the combination of condition and intensity that yields the greatest SWL; neither the condition nor the pacing on their own result in similar effects. For ease of readability and for incorporating participant feedback, we break the findings down per condition (design: coins versus paper) and intensity (spread versus condensed).

**Table 2:** Means (SDs) satisfaction with life (SWL) at six measure moments

SWLS	A1 coin; spread	A2 coin; condensed	B1 paper; spread	B2 paper; condensed	Control group	Cronbach’s alpha
Pre-study	5.25 (1.08)	4.87 (0.96)	5.27 (0.88)	4.75 (1.29)	4.57 (1.24)	0.804
Week 1 (on day 7)	5.00(1.20)	5.01 (1.16)	4.91 (1.45)	4.51 (1.65)	4.75 (1.48)	0.853
Week 2 (on day 14)	5.05 (1.05)	4.92 (1.12)	4.98 (1.12)	4.35 (1.60)	4.98 (1.23)	0.815
Week 3 (on day 21)	5.49 (0.77)	4.81 (0.84)	4.81 (1.28)	4.61 (1.68)	4.95 (1.28)	0.859
Week 4 (on day 28)	5.66 (1.17)	5.19 (0.76)	4.91 (1.17)	4.72 (1.73)	4.79 (1.53)	0.884
Week 5 (on day 35)	5.39 (1.44)	4.81 (1.65)	4.91 (1.18)	4.67 (2.13)	4.83 (1.72)	0.889

### Design: Coins Versus Paper

Participants who were given the tasks on coins reported higher SWL levels than those who were given the tasks on paper, which applied to those in both the spread and condensed groups (see Table 2). Feedback given by participants in the post-research questionnaire is congruent with these findings. All participants in the Coin condition who gave feedback were positive about the task being delivered on a key ring coin. Some mentioned that it was exciting to be reminded to do the task whenever they were using their keys. Others mentioned that the coins helped them to put their hearts into the project, trying their best to finish the tasks. Some mentioned that they did not attach the coin to their key ring, but kept it in another visible place (e.g. one stuck the coin to his laptop, another put it in her wallet). Several participants mentioned that they had given (or wanted to give) some of these coins to friends or family members in order to share their pleasant experiences. In addition, several participants in the Paper condition mentioned that they found it hard to remember their tasks and had to develop a procedure to remind themselves, like writing the task in a notebook or creating a note on their phone.

### Intensity: Spread versus Condensed

Participants who performed one task a day reported higher SWL levels than those who did all six tasks on one day, which applied to those in both the Coin and Paper conditions (see Table 2). The feedback given by participants in the post-research questionnaire provides some insight into how they experienced having to do what amounted to a week’s worth of tasks in 1 day. Several explicitly mentioned that they found it very challenging to do six tasks in 1 day. They mentioned two reasons: firstly, they did not have much time to prepare, because they were informed about the tasks on the evening before the day they did all six activities; and secondly, they found it difficult to take the time away from their other duties to perform all six tasks. Several respondents mentioned that the challenges caused them to experience negative emotions like stress or dissatisfaction. Conversely, several participants who were asked to do one task a day mentioned that they experienced positive emotions like excitement and enjoyment during the six-week period.

### Intentions for Post-Study Usage of Coins

In the post-study questionnaire, respondents in the Coin condition were asked if they had kept the coins, and, if so, what they did (or intended to do) with them. All 20 respondents who answered this question mentioned that they had kept the coins. Most of them mentioned that the coins would serve as a reminder of their experiences (e.g. ‘*I keep them as reminders and inspiration to do something just for fun’*; ‘*I kept them to remember them*’) or because they want to reuse them in the future (e.g. ‘*I want to reuse them next year, after the summer holiday’*, ‘*I wish to use them some time in future. Periodically, I take stock of how things are going on in my life. In one of those reflections, I may start using the key ring coins in some way again.*’). Some mentioned that they had kept a selection of coins that had a special meaning for them (e.g. ‘*I kept the ones that inspired me’*, ‘*I kept only the key ring coins that reminded me of tasks that, after completing them, gave me a very satisfied feeling or really changed something in my life. For example, the key ring coin linked to the task in which I had to admit my feelings to someone; this led to me and my boyfriend getting together, something I am very happy about!*’). Several mentioned that they planned to give them to other people (e.g. ‘*I will give them to someone else in the future’*; ‘*I think they will be a good present.*’). Others mentioned that they did not have a clear reason for keeping them (e.g. ‘*I kept them, but I don’t really know why; I think as some sort of remembrance of the tasks I did*’, or ‘*I just could not throw them away, but there is not really a reason for that.*’) or because they simply liked the design (e.g. ‘*I like their design, and they are colourful. I don’t want to throw them away.*’).

## Conclusions

This study examined the happiness-effect of using tangible coins for communicating self-administered happiness activities. It was hypothesized that TinyTask can contribute to user life satisfaction, and that this contribution is caused not only by the assignments (happiness-enhancing activities) but also by the physical key ring coins used in the TinyTask procedure. In a six-week long study, we found a three-way interaction effect between design (tasks on coins versus tasks written on paper), intensity of performance (six tasks a week: one task a day versus six tasks on 1 day), and time. More specifically, the study indicated that happiness-enhancing activities presented through a tangible coin lead to greater SWL levels (than when presented on paper), and that this effect increases over time (RQ2). Respondents kept the coins as a reminder of their experiences, to explore the possibility of doing the activities again, or to give them to someone as a present. Also, the data showed that these activities had the strongest influence on SWL if they were spread out during the week instead of being intensively performed on a single day (RQ3). Furthermore, and consistent with previous literature, we found that performing happiness-enhancing activities increases people’s SWL levels as compared with a control group (RQ1).

## General Discussion

In 2000, Ken Sheldon and his colleagues published a ‘Positive Psychology Manifesto’, in which they defined positive psychology and explained its objectives, applications, and implementation goals. In the final part of the document, they discussed their vision of what the optimal conditions are for the flourishing of positive psychology itself. One piece of advice they offered was to produce ‘useful and inspiring products, such as articles, books, and effective interventions’ (Sheldon et al. 2000, p. 1). This advice intends to explicitly promote spreading positive psychological principles and perspectives to a broad audience. Many of the most influential positive psychologists have written books that aim to reach this broad audience (e.g. Lyubomirsky 2013; Seligman 2011). The limitation of these self-help books, however, is that they reach only a select group of people—those who love to read (Wilson and Cash 2000). Moreover, it is not clear how effective these books are. In a review paper, Bergsma (2007) concluded that although there is some evidence that reading problem-focused self-help books is useful for people with specific problems, there is no evidence for the effectiveness of reading growth-oriented books. In search of more efficient alternatives, interactive technology has been discovered as a means to make positive psychology known to a greater number of people (Sander 2011). Bolier (2015) showed that online interventions provide scholars and practitioners working in positive psychology with many opportunities to develop enjoyable, engaging interventions that are scalable. Likewise, Morris and Picard (2014) indicated that BITs offer exciting new ways to disseminate PPIs. Self-guided BITs can reach populations that may not have the resources or motivation to pursue traditional, therapist-led interventions (Schueller et al. 2013; Mohr et al. 2013). In that sense, new technologies can ‘democratize’ positive psychology (Sander 2011). Even though they are promising, currently available self-guided BITs are not always effective (Sin and Lyubomirsky 2009; Bolier 2015). One important restraining factor is a lack of user engagement and motivation (Morris and Picard 2014). The success of PPIs depends on whether users put forth sufficient effort when doing the activities (Lyubomirsky et al. 2011), and the motivation to put forth this effort quickly drops when computer-based interventions are self-administered (Christensen et al. 2009; Eysenbach 2005). Morris and Picard (2014) and Schueller et al. (2013) therefore proposed that careful attention should be paid to the design of BITs: when poorly designed and unengaging, they will be of little benefit for the wider population. In this paper we demonstrated that one promising opportunity for improving the design of self-guided BITs is to include *tangibility*. Tangible products form the material context of our daily lives. They affect us; they inspire us, they frustrate, delight, and annoy us (Desmet 2012), and they can demotivate or inhibit us, but also uplift and enable us (Manzini 2005; Morelli 2007). While all currently available BITs rely on screens (computers, tablets, phones), our study demonstrates that tangible products represent an untapped potential for bringing positive psychology to the everyday lives of many people.

The ‘activity advice’ in positive psychology and the ‘experience recommendation’ in consumer research equally suggest that it does not pay off to seek happiness in material objects (Nicolao et al. 2009; Van Boven 2005; Dunn et al. 2011). Indeed, there is an impressive amount of evidence that happiness achieved with newly purchased consumer products quickly wears off (Patterson and Biswas-Diener 2012; Carter and Gilovich 2012). However, this shows only one side of the coin. Our study demonstrates that material objects can be designed deliberately to facilitate and enable meaningful activities and experiences that increase happiness. Thus, rather than being objects of happiness themselves, tangible objects can contribute to our happiness by helping us to engage in meaningful activities, by serving as reminders of our past experiences, and by serving as tokens that can be used to share these experiences with others. Design has gained a great deal from working with psychologists. These collaborations can lead to products and services that evoke experiences that are enjoyable and meaningful and that contribute to well-being (for examples, see Desmet and Pohlmeyer 2013). Likewise, psychology can benefit greatly from collaborating with designers. Schueller et al. (2013) proposed that in order to create effective BITs, psychologists, by necessity, must team with developers, such as technologists, engineers, and computer scientists. These authors stressed that this teaming up should involve active collaboration that spans the development, implementation, and dissemination of research. We foresee that such multidisciplinary collaborations will stimulate the development of a varied repertoire of (online and offline) technologies that can empower a broad audience to integrate principles of positive psychology in their everyday interactions. Design focuses by nature on daily practices and routines, and designers are trained to create solutions that are relevant and meaningful in these practices. Whereas traditional (screen-based) BITs can be perceived as demanding or challenging, these new solutions might support people in adopting a more natural and individual approach to self-guided personal growth.

The results presented here were robust, but it should be noted that our argument in this paper is based on but a single study. Moreover, given the relatively small sample size of the study, one should be cautious about the generalizability of the three-way interaction. Therefore, we invite initiatives aiming to replicate the results across samples, stimuli, and situations. We especially encourage research that further investigates the antecedents, nature, and consequences of the interplay between tangible and intangible components in offerings across contexts. An interesting additional question to be addressed by future research is why tangible elements enhance the effects of happiness-enhancing activities. Several explanations are possible: it could be that tangibility helps people to recall the experience (as was mentioned by some of the respondents), or that thanks to the tangibility the experience becomes deeper and more profound. Although the focus of our study was on the use of tangible coins, it should be explored how tangible and digital means can be combined to increase efficacy and effectiveness.

Although a six-week study qualifies as longitudinal research, the long-term effects of TinyTask (or other forms of positive activity interventions) on one’s chronic happiness level are not yet known. Are these effects sustainable, or will they wear off after several months or years? Our study results show that the happiness effect of TinyTask increases over time, but we also found a dampening effect at the end of the study. This effect may have been stimulated by methodological causes. For example, participants may have gotten tired of having to fill out daily questionnaires, or the fact that they did not engage in the activities on their own initiative but because it was part of a study may also have had a negative effect on their intrinsic motivation and subsequent SWL measures. However, the dampening effect may also have been influenced by the TinyTask design itself. Perhaps respondents lost some of their interest once the novelty effect of the intervention had worn off. The colourful coins may not have a long-lasting motivating effect. The decreasing motivation to commit to interventions is a general challenge in intervention technology, and our study does not provide indications that the TinyTask design addressed this challenge. It is therefore interesting to explore the optimal conditions for stimulating long-term usage motivations for initiatives like TinyTask. For example, coins could be administered for some months every year to keep the experience fresh, or perhaps the frequency of coins could vary or gradually deintensify after longer usage. Another opportunity would be to explore how the design can facilitate experiences of relatedness over longer usage periods, which (next to autonomy and competence) can stimulate one’s intrinsic motivation (Ryan and Deci 2000) to engage in the activities. These design explorations may contribute to the development of sustainable behavioural intervention technologies with longer-term contributions to user happiness.

## Appendix 1

Overview of 50 tasks used in main study.

1. *Acts of kindness*/Perform acts of kindness to three strangers in 1 day. Give up your place in a queue to someone behind you, give someone your seat on the bus, etc.
2. *Barefoot*/Walk around at work without shoes for 1 h. You may also go outside if you dare.
3. *Be a reporter*/Be a reporter for a day and write a short news report about something good that has happened to you. Print the report and hang it on your wall or send it to a friend.
4. *Break it down*/To make progress towards a large goal (such as finding a new job or home); break it down into smaller steps. Plan how and when you will complete these small steps.
5. *Clean up*/Clean something that has been bothering you; e.g. your mouse, keypad, door handles, mirror, windows, etc.
6. *Donate*/Find a product (e.g. a piece of clothing or a book) you don’t use anymore and donate it to a friend, neighbour, or the local charity shop. Do it today or schedule it in your agenda.
7. *Early night*/Go to bed an hour earlier than you normally would.
8. *Family tree*/Draw a family tree. Use your own knowledge as a starting point, you can ask a relative to help you out.
9. *Favourite song*/Play your favourite song out loud, sing and dance along with it.
10. *Forgive*/Forgive someone by writing a statement of forgiveness. Keep it private for a week. You may then keep the statement to yourself, burn it, or present it to the one you’ve forgiven, do whatever you feel is best.
11. *Get involved*/Get involved in a movement or a cause that is new to you. You can participate in a flash mob, follow an interesting blog or join an evening class.
12. *Good ol’ times*/Find a picture that brings back fond memories, print it out and hang it on the wall, or set it as your computer background image.
13. *Green light*/Obey all the traffic signs and regulations for 1 day.
14. *Guerrilla florist*/Be a guerrilla florist by secretly planting some flowers in your neighbourhood. Don’t forget to look after them after they’re planted.
15. *Happy moments*/Think about three truly happy moments in your past. Write them down on cards, and hang them on the wall or on the fridge.
16. *Healthy meal*/Prepare a truly healthy meal for yourself and take the time to enjoy it.
17. *In good company*/Write a thank you letter or e-mail to a company whom you think delivers a good service, and send it.
18. *Inspiring stories*/Read or watch one inspiring story (e.g. http://www.ted.com) about people who managed to make their dreams come true due to their persistence and high spirit. You can also ask a friend who inspires him or her.
19. *Keep a tally*/Choose something you like (small dogs, red cars, men with moustaches), and keep a tally: For 1 day, count how many times you encounter the thing you’ve chosen. You can write it down in a notebook or on the back of your hand.
20. *Kind words*/Pay at least three people you encounter today a genuine compliment.
21. *Learn a language*/Learn a new language for 1 day. Learn ten new words, write them down and hang them on your wall.
22. *Listen up*/Be a listening ear to a friend who needs you, offer your shoulder if he/she needs it.
23. *Look up*/Look up (at the sky, the clouds, and high buildings), discover something new and make a drawing or photo of it.
24. *New friend*/Make a new friend by talking to someone you don’t know very well, and find out something interesting about his/her life.
25. *New room*/Redecorate your room by shifting your furniture, or buy a new bed sheet, pillowcase or poster.
26. *New way home*/Travel home today using a completely new route or use a different means of transportation.
27. *Next door*/Post a kind letter to a neighbour you haven’t met to introduce yourself. Attach your address so they can write back.
28. *Pick up litter*/Pick up litter from the street you live in, to benefit you and your neighbours.
29. *Plan a date*/Plan a date with someone you haven’t spoken with for a long time.
30. *Plan a holiday*/Plan a holiday with a friend. Your destination can be expensive or cheap, nearby or far away, depending on your possibilities.
31. *Positive future*/Make a drawing of your best possible future to visualize your dream situation: where would you live, what would you do for a job?
32. *Prepare a meal*/Prepare a meal for someone to thank him/her. Don’t forget to actually say thanks.
33. *Project*/Write down a plan for a small project to which you want to commit, but haven’t found the time for.
34. *Read it? Share it!*/Wrap up any recent magazine issues you’ve already read, and post them at neighbour’s houses. Don’t use issues that are dirty or old.
35. *Repair*/Find a problem in your house and solve it. Changing a bulb, fixing a hole in the wall; anything goes!
36. *Seclusion*/Find a place in nature that is secluded from traffic and buildings. Listen to the birds, wind, and surroundings.
37. *Share a joke*/Share a joke with at least one person today.
38. *Share your goals*/Share your goals with your friends or family, so they can remind or motivate you to pursue your goals.
39. *Share your insight*/Share your knowledge about a topic of your expertise. For instance; teach a friend how to use a camera. You can also blog about your knowledge or make a small instruction pamphlet.
40. *Small stroll*/Take a walk for 10 min by yourself, on a moment that suits you.
41. *Smiling mirror*/Smile to yourself in the mirror and draw your smile on it with non-permanent marker or lipstick, so you will run into your own smiling face again.
42. *Stair master*/Avoid all elevators and escalators, and take only the stairs for 1 day.
43. *Start saving*/Acquire a piggy bank and save money to buy something you really want.
44. *Stretch*/Stretch in the morning or evening for 5–15 min.
45. *Surprise yourself*/Write down your qualities or positive characteristics on post-its. Hide them somewhere in your home so you will face them later again.
46. *Sweet notes*/Leave sweet messages for your family or colleagues at random places (e.g. in someone’s’ bag, or at someone’s desk) to surprise them.
47. *Take notice*/Just breathe and take notice of your body and feelings for 5–15 min.
48. *Time out*/Take a time out. For 15 min, don’t communicate, listen to music or watch the TV.
49. *Treat*/Give a good friend or colleague a treat with some sweets or a cup of coffee.
50. *Write a letter*/Write a letter to thank someone you’ve never properly thanked and send it.

## References

[CR2] Bergsma A (2007). Do self-help books help?. J Happiness Stud.

[CR3] Bolier L (2015). Positive psychology online: using the internet to promote flourishing on a large scale (Unpublished doctoral dissertation).

[CR4] Bolier L, Haverman M, Westerhof GJ, Riper H, Smit F, Bohlmeyer E (2013). Positive psychology interventions: a meta-analysis of randomized controlled studies. BMC Public Health.

[CR5] Calvo RA, Peters D (2014). Positive computing: technology for well-being and human potential.

[CR6] Carter TJ, Gilovich T (2012). I am what I do, not what I have: the differential centrality of experiential and material purchases to the self. J Pers Soc Psychol.

[CR7] Christensen H, Griffiths KM, Farrer L (2009). Adherence in internet interventions for anxiety and depression. J Med Internet Res.

[CR8] Consolvo S, McDonald DW, Landay JA, Konstan JA, Chi E, Höök K (2009). Theory-driven design strategies for technologies that support behavior change in everyday life. Proceedings of the SIGCHI Conference on Human Factors in Computing Systems.

[CR9] Deci EL, Ryan RM (1985). Intrinsic motivation and self-determination in human behavior.

[CR10] Desmet PMA (2012). Faces of product pleasure: 25 Positive emotions in human-product interactions. Int J Des.

[CR11] Desmet PMA, Pohlmeyer AE (2013). Positive design: an introduction to design for subjective well-being. Int J Des.

[CR12] Diener E (1999). Subjective well-being: three decades of progress. Psychol Bull.

[CR13] Diener E, Emmons RA, Larsen RJ, Griffin S (1985). The satisfaction with life scale. J Pers Assess.

[CR14] Dunn EW, Gilbert DT, Wilson TD (2011). If money doesn’t make you happy, then you probably aren’t spending it right. J Consum Psychol.

[CR15] Eysenbach G (2005). The law of attrition. J Med Internet Res.

[CR16] Fogg BJ. A behavior model for persuasive design. In: Chatterjee A, Dev P, editors. Persuasive 2009. Proceedings of the 4th international conference on persuasive technology. New York: ACM; 2009. Article no. 40.

[CR17] Hassenzahl M (2010). Experience design: technology for all the right reasons. Synth Lect Hum Cent Inform.

[CR18] Layous K, Lyubomirsky S, Gruber J, Moscowitz J (2014). The how, why, what, when, and who of happiness: mechanisms underlying the success of positive interventions. Positive emotion: integrating the light sides and dark sides.

[CR19] Lyubomirsky S (2001). Why are some people happier than others?: the role of cognitive and motivational processes in well-being. Am Psychol.

[CR20] Lyubomirsky S (2008). The how of happiness: a new approach to getting the life you want.

[CR21] Lyubomirsky S (2013). The myths of happiness: what should make you happy, but doesn’t, what shouldn’t make you happy, but does.

[CR22] Lyubomirsky S, Dickerhoof R, Boehm JK, Sheldon KM (2011). Becoming happier takes both a will and a proper way: an experimental longitudinal intervention to boost well-being. Emotion.

[CR23] Lyubomirsky S, Sheldon KM, Schkade D (2005). Pursuing happiness: the architecture of sustainable change. Rev Gener Psychol.

[CR24] Manzini E (2005). Enabling solutions for creative communities. Designmatters.

[CR25] McGonical J (2011). Reality is broken: why games make us better and how they can change te world.

[CR26] Mohr DC, Burns MN, Schueller SM, Clarke G, Klinkman M (2013). Behavioral intervention technologies: evidence review and recommendations for future research in mental health. Gen Hosp Psychiatry.

[CR27] Montague EN, Kleiner BM, Winchester WW (2009). Empirically understanding trust in medical technology. Int J Ind Ergon.

[CR28] Morelli N (2007). Social innovation and new industrial contexts: can designers “industrialize” socially responsible solutions?. Des Issues.

[CR29] Morris RR, Picard R (2014). Crowd-powered positive psychological interventions. J Posit Psychol.

[CR30] Muñoz RF (2010). Using evidence-based internet interventions to reduce health disparities worldwide. J Med Internet Res.

[CR31] Nicolao L, Irwin JR, Goodman JK (2009). Happiness for sale: do experiential purchases make consumers happier than material purchases?. J Consum Res.

[CR32] Parks AC, Joseph S (2015). Putting positive psychology into practice via self-help. Positive psychology in practice: promoting human flourishing in work, health, education, and everyday life.

[CR33] Parks AC, Biswas-Diener R, Kashdan T, Ciarrochi J (2013). Positive interventions: past, present and future. Bridging acceptance and commitment therapy and positive psychology: a practitioner’s guide to a unifying framework.

[CR34] Parks AC, Szanto RK (2013). Assessing the efficacy and effectiveness of a positive psychology-based self-help book. Terapia Psicológica.

[CR35] Parks AC, Schueller SM, Tasimi A, David S, Boniwell I, Ayers AC (2012). Increasing happiness in the general population: empirically supported self-help. Oxford handbook of happiness.

[CR36] Patterson L, Biswas-Diener R, Brey P, Briggle A, Spence E (2012). Consuming happiness. The good life in a technological age.

[CR37] Pavot WG, Diener E, Colvin CR, Sandvik E (1991). Further validation of the satisfaction with life scale: evidence for the cross-method convergence of well-being measures. J Pers Assess.

[CR100] Ruitenberg HP. Designing for subjective well-being. Unpublished master’s thesis, Delft University of Technology, The Netherlands.

[CR38] Ryan RM, Deci EL (2000). Intrinsic and extrinsic motivations: classic definitions and new directions. Contemp Educ Psychol.

[CR39] Sander T, Biswas-Diener R (2011). Positive computing. Positive psychology as social change.

[CR40] Schueller SM, Parks AC (2014). The science of self-help: translating positive psychology research into increased individual happiness. Eur Psychol.

[CR41] Schueller SM, Muñoz RF, Mohr DC (2013). Realizing the potential of behavioral intervention technologies. Curr Dir Psychol Sci.

[CR42] Seligman MEP (2011). Flourish.

[CR43] Sheldon KM, Elliot AJ (1998). Not all personal goals are personal: comparing autonomous and controlled reasons for goals as predictors of effort and attainment. Pers Soc Psychol Bull..

[CR44] Sheldon KM, Lyubomirsky S (2006). Achieving sustainable gains in happiness: change your actions, not your circumstances. J Happiness Stud.

[CR45] Sheldon KM, Fredrickson B, Rathunde K, Csikszentmihalyi M, Haidt J. Positive psychology manifesto. 2000. http://www.ppc.sas.upenn.edu/akumalmanifesto.htm. Accessed 9 May 2015.

[CR46] Sin NL, Lyubomirsky S (2009). Enhancing well-being and alleviating depressive symptoms with positive psychology interventions: a practice-friendly meta-analysis. J Clin Psychol.

[CR47] Van Boven L (2005). Experientialism, materialism, and the pursuit of happiness. Rev Gener Psychol.

[CR48] Wilson DM, Cash TF (2000). Who reads self-help books? Development and validation of the self-help reading attitudes survey. Personal Individ Differ.

